# Integrated metabolomic insights into the mechanism of *Bacillus coagulans* in enhancing intestinal recovery following gynecological laparoscopic surgery: a randomized clinical trial

**DOI:** 10.3389/fimmu.2025.1630447

**Published:** 2025-09-26

**Authors:** Juan Xu, Lingjun Dong, Chunqi Feng, Zhaobo Guan, Jingmin Zhang, Niuniu Bai, Zhanqin Niu, Weihong Chen, Weiqi Gao

**Affiliations:** ^1^ Third Hospital of Shanxi Medical University, Shanxi Bethune Hospital, Shanxi Academy of Medical Sciences, Tongji Shanxi Hospital, Taiyuan, China; ^2^ School of Pharmacy, Shanxi Medical University, Taiyuan, China; ^3^ Department of Pharmacy, Changzhi People’s Hospital, The Affiliated Hospital of Shanxi Medical University, Changzhi, China; ^4^ Shanxi Academy of Advanced Research and Innovation (SAARI), Taiyuan, China

**Keywords:** *Bacillus coagulans*, gynecological laparoscopy, intestinal function recovery, metabolomics, serotonin

## Abstract

**Objective:**

To evaluate the efficacy and mechanistic underpinnings of live *Bacillus coagulans* tablets in accelerating recovery of intestinal function after gynecologic laparoscopic surgery.

**Methods:**

A randomized, double-blind, placebo-controlled trial included 115 patients undergoing gynecological laparoscopy, assigned to placebo control (PC, *n* = 39), conventional dose (CD, *n* = 38), and high dose (HD, *n* = 38) groups. Clinical recovery was assessed by timing of first postoperative bowel movement and gastrointestinal adverse reactions. Plasma levels of motilin (MTL) and serotonin (5-HT) were measured using ELISA. Plasma metabolite profiles were analyzed via metabolomics to elucidate treatment-related metabolic pathways.

**Results:**

Treatment groups (CD and HD) exhibited significantly reduced time to first postoperative defecation and fewer gastrointestinal adverse reactions compared to PC (*P* < 0.05), without significant differences between CD and HD groups. Plasma MTL and 5-HT levels significantly increased postoperatively in the treatment groups compared with PC (*P* < 0.05), without dose-dependent differences. Metabolomics identified 50 and 73 differential metabolites in CD and HD groups post-treatment, respectively, enriched mainly in pathways involving tryptophan, tyrosine, histidine, kynurenine, primary bile acids, and short-chain fatty acids.

**Conclusion:**

Live *Bacillus coagulans* tablets effectively promoted postoperative intestinal recovery in patients undergoing gynecological laparoscopy. The mechanisms likely involve enhanced secretion of MTL and 5-HT, coupled with regulation of key metabolic pathways including tryptophan, tyrosine, kynurenine, histidine metabolism, primary bile acid synthesis, and SCFA metabolism. This study provides insights into probiotics’ potential to improve postoperative gastrointestinal outcomes.

**Clinical trial registration:**

https://www.chictr.org.cn/, identifier ChiCTR2400079659.

## 1 Introduction

Postoperative gastrointestinal dysfunction (POGD) is a common complication after abdominal surgery (1). It is typically characterized by delayed gastric emptying and partial intestinal obstruction. The occurrence of POGD is often linked to multiple factors, such as surgical trauma, anesthesia methods, electrolyte imbalance, intra−abdominal inflammation, residual blood, and mechanical irritation from drainage tubes. Collectively, these noxious stimuli disturb gut motility in part by upsetting the intestinal microbiota and mucosal immune balance, making microbiome−targeted strategies—such as probiotics—a biologically rational therapeutic option. Clinical symptoms usually include abdominal distension, abdominal pain, nausea, vomiting, and cessation of bowel movements and defecation. These are inevitable physiological reactions to surgical and anesthetic stress (2). Main pathophysiological changes include intestinal dilatation, fluid and electrolyte imbalance, infection, and toxaemia (3). Although the incidence and severity of POGD after laparoscopic surgery are less than those after open surgery (4), recovery of gastrointestinal function remains crucial for perioperative rehabilitation and prognosis. Currently, alleviating POGD involves reducing surgical trauma, preventing inflammatory reactions, minimizing opioid use, encouraging postoperative chewing, early mobilization, early feeding (5), coffee intake (6), chewing gum (7), and probiotics (8, 9). However, these methods are not universally effective, their results vary, and standardized implementation remains limited. Commercially available *Bacillus coagulans* tablets (Shuangshubao) were therefore selected for investigation. Ja Park et al. (10) recently demonstrated that peri−operative probiotic administration reduced postoperative flatulence, modified intestinal microbiota, and attenuated inflammatory markers, further justifying our focus on probiotic therapy. Its primary effect is to regulate human intestinal flora, and it uniquely possesses water−retention and laxative functions among probiotics (11, 12). Previous studies indicate *Bacillus coagulans* can enhance intestinal peristalsis and gastric motility, thus restoring gastrointestinal function (13, 14).

Motilin (MTL) is an endogenous peptide hormone secreted by the intestines, mainly promoting gastrointestinal motility. Specifically, it enhances the third phase of interdigestive migrating motor complexes (15). However, the relationship between probiotics and plasma MTL levels remains unclear. Serotonin (5-HT), a neurotransmitter synthesized endogenously with hormone-like properties, approximately 95% of 5-HT is produced in the gut. Connections between gut microbiota metabolites, such as short-chain fatty acids (SCFAs), and 5-HT levels are established, with probiotics commonly employed to regulate 5-HT concentrations (16). Variations in 5-HT may influence gut motility through the gut-brain axis (17). However, few studies have explored the impact of 5-HT on the gut microbiota. Gut microbiota and the host’s 5-HT system communicate bidirectionally, maintaining intestinal homeostasis and overall health.

Previously, our group demonstrated that *Bacillus coagulans* tablets TBC169 effectively promoted intestinal gas passage in postoperative gynecologic abdominal surgery patients, including open and laparoscopic procedures (18). However, its precise mechanism remains unclear. Thus, this study aimed to assess the efficacy and safety of *Bacillus coagulans* tablets in promoting postoperative intestinal recovery in gynecologic laparoscopic surgery patients. Beyond clinical endpoints, we integrate targeted hormonal readouts with UHPLC-MS-based plasma metabolomics to map tryptophan/kynurenine (Kyn), bile-acid and short-chain-fatty-acid pathways, thereby providing mechanism-resolved and dose-sensitive signatures of probiotic action.

## 2 Materials and methods

### 2.1 Study design

#### 2.1.1 Study population

This randomized, double-blind, placebo-controlled clinical trial was conducted at the Department of Obstetrics and Gynecology in Bethune Hospital in Shanxi, China, from March to August 2024. A total of 115 female patients undergoing elective gynecological laparoscopic surgery (grade III or higher) were enrolled. The sample size was calculated using PASS 11 software based on pilot data, aiming at a 90% power and an *α*-level of 0.05, with an expected dropout rate of 5%. Consequently, approximately 120 participants were initially planned to be recruited to achieve statistical robustness.

#### 2.1.2 Inclusion, exclusion, and dropout criteria

Eligible patients included females aged 18–65 years, scheduled for laparoscopic surgery lasting 1–4 hours, who voluntarily provided written informed consent. Participants with cognitive disorders, such as depression or Alzheimer’s disease, a history of abdominal surgery or probiotic allergies, or intraoperative blood loss exceeding 400 mL were excluded. Patients concurrently enrolled in other clinical trials or who had participated within three months were also excluded. Criteria for trial dropout included serious adverse reactions, voluntary withdrawal by the participant, or inability to adhere to trial medication protocols.

#### 2.1.3 Treatment protocol and medications

Participants were randomly assigned into three groups: placebo control (PC, *n* = 39), conventional dose (CD, *n* = 38), and high dose (HD, *n* = 38). All patients underwent uniform preoperative bowel cleansing. The PC group received six placebo tablets orally at 21:00 the night before surgery and every 8 h postoperatively until first anal defecation. Patients in the CD group took six tablets preoperatively, followed by three probiotic tablets combined with three placebo tablets every 8 h postoperatively until first defecation. The HD group received six probiotic tablets preoperatively and continued with six probiotic tablets every 8 h after surgery until the same endpoint.

The probiotic preparation, *Bacillus coagulans* tablets (Shuangshubao, Qingdao Donghai Pharmaceutical Co., Ltd), contains 350 mg per tablet with ≥1.75×10^7 colony-forming units (CFU) live *Bacillus coagulans* TBC169. Placebo tablets were identical in appearance, texture, and taste but contained no live bacteria. For dose transparency, the CD group received 6 active tablets preoperatively (≥1.05×10^8 CFU), then 3 active tablets every 8 h postoperatively (≥5.25×10^7 CFU per administration; approximately ≥1.58×10^8 CFU/day until first defecation). The HD group received 6 active tablets preoperatively (≥1.05×10^8 CFU), then 6 active tablets every 8 h postoperatively (≥1.05×10^8 CFU per administration; approximately ≥3.15×10^8 CFU/day until first defecation). A high-dose arm was included to explore potential dose–response within an established safety margin and to evaluate whether the labeled (conventional) dose achieves comparable clinical benefit with lower pill burden and cost. If no defecation occurred within 72 hours postoperatively, attending physicians applied alternative methods to facilitate bowel movement.

### 2.2 Outcome assessment and analytical methods

#### 2.2.1 Randomization and blinding procedures

Eligible participants were randomly allocated in a 1:1:1 ratio into PC, CD, and HD groups. Randomization was performed using computer-generated numbers. To maintain double-blind conditions, research drugs and placebo tablets were packaged by dedicated personnel into identical black-sealed bags. These packages were shuffled and distributed into three indistinguishable boxes labeled A, B, and C. Only the packaging personnel knew the exact treatment allocation corresponding to each box. Patients, clinicians, and researchers remained blinded throughout the trial. Patient enrollment followed a predetermined sequential order based on the randomized list. After trial completion and clinical data collection, a structured unblinding process was conducted by consulting packaging personnel to disclose group assignments.

#### 2.2.2 Clinical outcomes and data recording

Demographic information (age, BMI), surgical details (duration of anesthesia and surgery), and postoperative gastrointestinal function parameters were documented. The primary efficacy outcome was the interval between anesthesia recovery and first postoperative defecation. Secondary outcomes included incidence and severity of gastrointestinal adverse reactions, such as nausea, vomiting, bloating, and bowel obstruction. Safety evaluation involved monitoring probiotic-related adverse events, including systemic infections.

#### 2.2.3 Plasma collection and preparation

Patient plasma samples were collected preoperatively and immediately after first postoperative anal defecation. Blood (4 mL) was obtained from fasting patients, centrifuged (1769×g, 15 min, 4°C), and plasma was aliquoted and stored at -80°C for subsequent biochemical and metabolomic assays.

#### 2.2.4 Measurement of plasma biochemical markers

Plasma concentrations of MTL and 5-HT were quantified using commercial ELISA kits (Jiangsu Enzyme Immunity Industry Co., Ltd.) according to manufacturer instructions. Assays were performed in duplicate on a 96-well plate format, with colorimetric detection by an Infinite M Nano 200 PRO microplate reader (Tecan, Switzerland). Assay precision was confirmed with intra-plate CV <5% and inter-plate CV <8%. Pre-specified exclusion and quality-control criteria were applied: hemolyzed specimens, insufficient blood volume for duplicate measurement, excessive duplicate variability, out-of-range signals not recoverable upon repeat, or any failure to meet QC requirements. Only QC-passing specimens were analyzed. Statistical procedures for all clinical and biomarker analyses are described in Section 2.2.9.

#### 2.2.5 Metabolomics sample handling

Plasma samples from participants with complete pre- and postoperative data were included in metabolomic analysis (23 samples from the CD group, 22 from the HD group). Plasma metabolites were extracted by protein precipitation. Specifically, 20 μL of plasma was mixed with 80 μL of chilled acetonitrile solution containing internal standards at the following working concentrations: Val-d8, 500 ng/mL; Phe-d5, 500 ng/mL; cholic acid-d4 (CA-d4), 200 ng/mL; LPC(17:0), 200 ng/mL; and heptadecanoic acid (C17:0, FFA IS), 500 ng/mL; yielding final concentrations in the extract equivalent to 0.8× the above values due to the 1:4 (plasma:IS solution) mixing ratio. Samples were incubated at 4°C for one hour, centrifuged (16798×g, 15 min, 4°C), and supernatants were filtered (0.22 μm). Quality control (QC) samples were prepared by pooling equal aliquots of each test sample to ensure data reliability (19).

#### 2.2.6 Liquid chromatography and mass spectrometry analysis

Chromatographic separation was achieved using a Waters Acquity UHPLC HSS T3 column (2.1×100 mm, 1.8 μm) maintained at 40 °C. The mobile phases consisted of 0.1% formic acid-water (phase A) and 0.1% formic acid-acetonitrile (phase B). A binary gradient program was used at a flow rate of 0.30 mL/min with a 2 μL injection volume as follows: 0.0–1.0 min, 15% B; 1.0–9.0 min, linear ramp to 95% B; 9.0–11.0 min, hold at 95% B (wash); 11.0–11.5 min, return to 15% B; after each run, the column was re-equilibrated at the initial conditions (15% B) for 5 min before the next injection. Mass spectrometric analysis employed UHPLC-QTRAP™ with electrospray ionization in positive and negative modes. Parameters included a source temperature of 550 °C, spray voltages of + 5500 V (positive) and -4500 V (negative), and gas pressures (GS1, GS2 at 55 psi, curtain gas at 30 psi).

#### 2.2.7 Metabolomics data analysis and interpretation

Raw mass spectrometry data were analyzed with SCIEX MultiQuant™ software (v3.0.3) for peak extraction and normalization by internal standards. Data quality and clustering were initially assessed by principal component analysis (PCA) using SIMCA 14.1 software. To identify significant metabolic changes between groups, orthogonal partial least squares discriminant analysis (OPLS-DA) was conducted, and model robustness was confirmed by permutation tests (200 iterations).

Differential metabolites were defined by VIP > 1.0 (from OPLS-DA), fold change > 1.2 or < 0.83, and Benjamini–Hochberg FDR-adjusted *P* < 0.05. Pathway enrichment analyses were performed using MetaboAnalyst 6.0 (https://www.metaboanalyst.ca/) to elucidate metabolic pathways potentially affected by *Bacillus coagulans* treatment. Kyoto Encyclopedia of Genes and Genomes (KEGG) database was utilized to provide metabolic context for identified pathways.

#### 2.2.8 Ethical approval and trial registration

The protocol complied with ethical guidelines of the Declaration of Helsinki and was approved by the Medical Ethics Committee of Shanxi Baiqun Hospital (approval number: YXLL-2023-275). The trial was prospectively registered with the China Clinical Trial Registry (ChiCTR2400079659). Written informed consent was obtained from all participants before enrollment.

#### 2.2.9 Statistical analysis

Analyses used IBM SPSS 26. Normality was tested by Shapiro–Wilk and homogeneity by Levene. Normal data: one-way ANOVA with Tukey’s HSD. Non-normal data: Kruskal–Wallis with Dunn’s *post-hoc* and Bonferroni adjustment. Within-group pre-post: paired t-tests or Wilcoxon. Postoperative hormones: ANCOVA with baseline and time from surgery to blood draw. Categorical data: chi-square or Fisher’s exact. Two-tailed P<0.05; Benjamini–Hochberg FDR.

## 3 Results

### 3.1 Clinical evaluation of *Bacillus coagulans* on postoperative intestinal function

#### 3.1.1 Patient demographic and clinical characteristics

**Figure 1:**
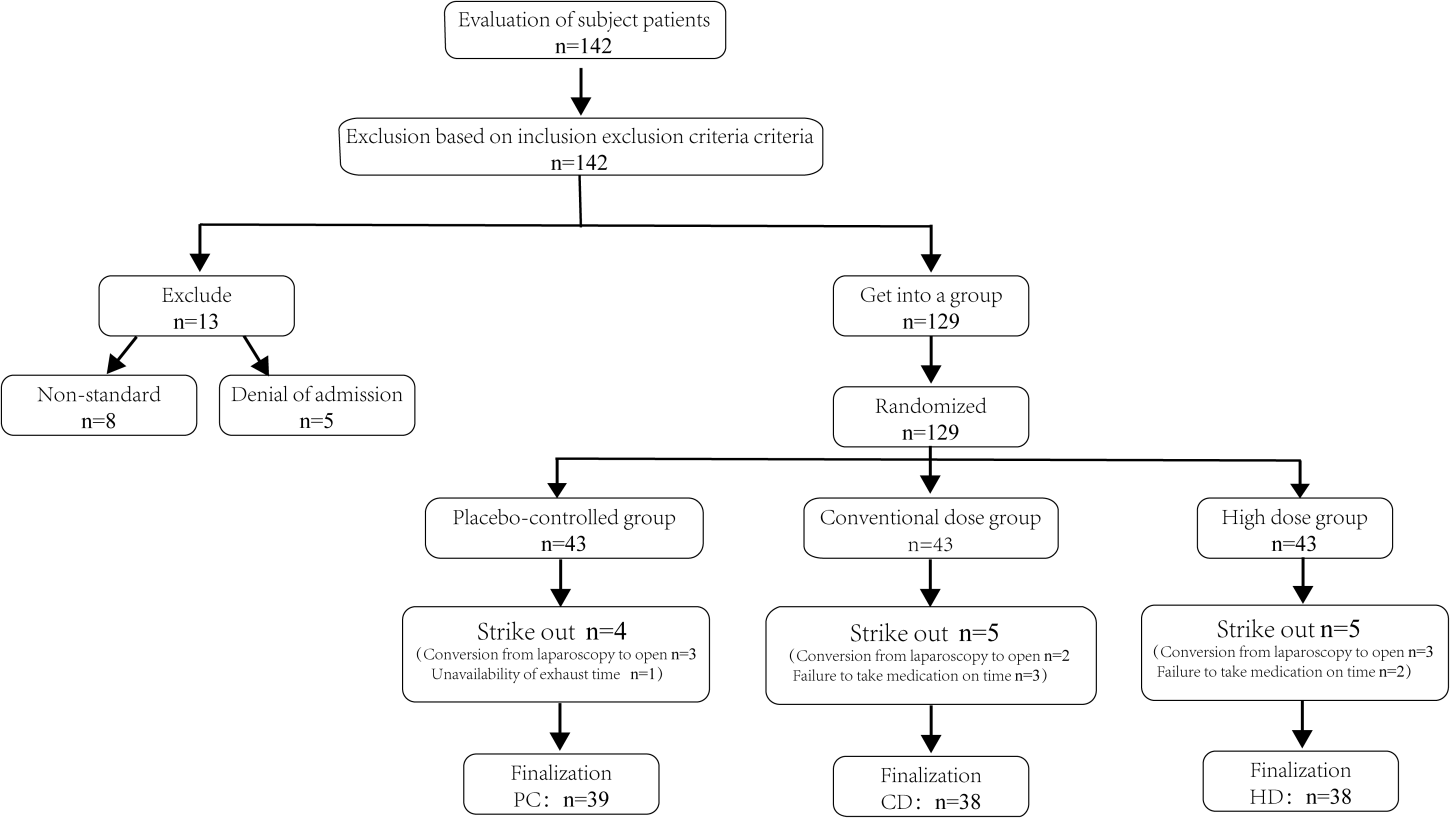
Flowchart for inclusion and screening of patients. PC, placebo control; CD, conventional dose; HD, high dose; n, number of participants.

Initially, 142 female patients undergoing elective gynecological laparoscopic surgery were screened for eligibility, and 129 met the inclusion criteria and were randomized. After subsequent dropouts, 115 participants completed the trial and were allocated to PC (*n* = 39), CD (*n* = 38), and HD (*n* = 38) (**Figure 1**). Baseline characteristics are summarized in the integrated table; groups were generally well balanced. Aside from a modest difference in height between PC and HD, there were no significant between-group differences in age, weight, BMI, length of surgery, or length of anesthesia (**Table 1**). This baseline comparability supports attributing postoperative differences to the effects of *Bacillus coagulans*.

**Table 1:** General clinical characteristics with pairwise comparisons among groups.

Clinical characteristic	PC (*n* = 39)	CD (*n* = 38)	HD (*n* = 38)	*P* (PC vs CD)	*P* (PC vs HD)	*P* (CD vs HD)
Age (years)	42.41 ± 9.70	40.74 ± 12.61	46.41 ± 10.71	0.540	0.091	0.073
Height (cm)	158.75 ± 5.51	160.87 ± 5.25	160.44 ± 3.84	0.120	0.026	0.730
Weight (kg)	63.96 ± 11.54	60.62 ± 12.10	62.97 ± 11.99	0.218	0.923	0.182
BMI	25.34 ± 4.11	23.42 ± 4.53	24.43 ± 4.31	0.219	0.669	0.121
Length of surgery (min)	108.76 ± 56.36	97.19 ± 40.32	112.41 ± 54.13	0.285	0.818	0.289
Length of anesthesia (min)	141.72 ± 58.32	129.94 ± 42.90	144.48 ± 59.90	0.324	0.885	0.395

Data are presented as mean ± standard deviation. *P* values are for pairwise comparisons between groups listed in the column headings.

PC, placebo control; CD, conventional-dose *Bacillus coagulans*; HD, high-dose *Bacillus coagulans*; BMI, body mass index; *n*, number of participants; min, minutes; *P*, *P* value.

#### 3.1.2 Effects of *Bacillus coagulans* on recovery of gastrointestinal function

Clinical effectiveness was primarily evaluated by the interval from anesthesia recovery to the first postoperative defecation (**Table 2**). Significant reductions were observed in the CD (808.90 ± 452.90 min) and HD groups (876.59 ± 551.10 min) compared with the PC group (1288.06 ± 890.21 min) (*P* = 0.020 and *P* = 0.016, respectively). However, no significant difference was identified between CD and HD groups (*P* = 0.610). This indicates that administration of *Bacillus coagulans* significantly accelerates postoperative bowel function recovery without clear dose-dependency within the tested ranges. Postoperative gastrointestinal adverse reactions are detailed in **Table 3**. Overall incidence rates of gastrointestinal adverse events were significantly lower in CD (13.15%) and HD (10.52%) groups compared to the PC group (38.46%, *P* = 0.007 and *P* < 0.001, respectively). The incidence rates for bloating, nausea, vomiting, and postoperative intestinal obstruction were lower in both treated groups, with statistical significance in total adverse reactions but not between CD and HD groups (*P* = 0.803). Importantly, no probiotic-related systemic adverse events were recorded, underscoring the safety profile of *Bacillus coagulans* tablets in postoperative patients.

**Table 2:** Time from anesthesia recovery to first postoperative bowel movement.

Measure	PC (*n* = 39)	CD (*n* = 38)	HD (*n* = 38)
Time from anesthesia recovery to first postoperative bowel movement (min)	1288.06 ± 890.21	808.90 ± 452.90	876.59 ± 551.10
*P* (PC *vs.* CD)	0.02		
*P* (PC *vs.* HD)	0.016		
*P* (CD *vs.* HD)	0.610		

PC, placebo control; CD, conventional-dose *Bacillus coagulans*; HD, high-dose *Bacillus coagulans*; *n*, number of participants; min, minutes; *P*, *P* value; *vs.*, versus.

**Table 3:** Results of statistical comparisons of safety endpoint indicator values for each group in PC, CD, and HD.

Projects	PC (*n* = 39)	CD (*n* = 38)	HD (*n* = 38)
Any gastrointestinal adverse event, *n* (%)	15 (38.46%)	5 (13.15%)	4 (10.52%)
Bloating, *n* (%)	6 (15.38%)	2 (5.26%)	2 (5.26%)
Nausea/vomiting, *n* (%)	8 (20.51%)	3 (7.89%)	2 (5.26%)
Postoperative intestinal obstruction, *n* (%)	1 (2.57%)	0 (0.00%)	0 (0.00%)
Results of the chi-square test (PC *vs* CD)	*χ*²=7.391, *P* = 0.007		
Results of the chi-square test (PC *vs* HD)	*χ*²=15.024, *P*<0.001		
Results of the chi-square test (CD *vs* HD)	*χ*²=0.062, *P* = 0.803		

PC, placebo control; CD, conventional-dose *Bacillus coagulans*; HD, high-dose *Bacillus coagulans*; *n*, number of participants; min, minutes; *P*, *P* value; *vs.*, versus; %, percentage; *χ²*, chi-square statistic.

### 3.2 Effects of *Bacillus coagulans* on plasma motilin (MTL) and serotonin (5-HT) levels

Following the prespecified QC and sampling procedures, the evaluable sample sizes were: preoperative—PC 21, CD 27, HD 26; postoperative—PC 38, CD 28, HD 24. The discrepancy reflects exclusion of hemolyzed/insufficient-volume or QC-failing specimens and a small number of missed preoperative draws due to operating-room logistics.

#### 3.2.1 Plasma MTL levels

**Figure 2:**
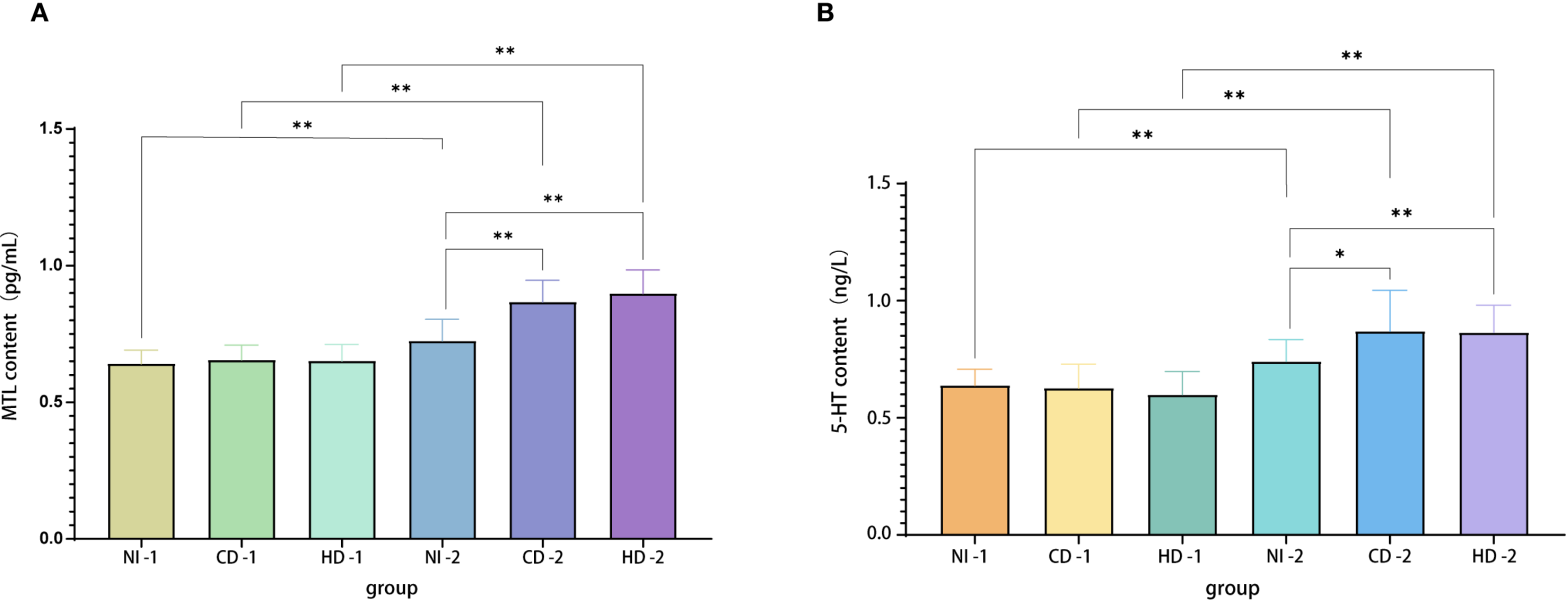
**(A)** MTL levels in plasma samples from each group. **(B)** 5-HT levels in plasma samples from each group. Evaluable N (pre/post): PC 21/38, CD 27/28, HD 26/24. Specimens with hemolysis, insufficient volume, or QC failure were excluded; a few preoperative draws were missed owing to operating-room scheduling. MTL, motilin; 5-HT, serotonin; PC, placebo control; CD, conventional dose; HD, high dose.

Plasma gastrointestinal hormone levels were assessed preoperatively and after the first postoperative defecation using ELISA. Intra-plate CVs were <5% and inter-plate CVs were <8%, validating assay precision. Preoperative MTL levels showed no statistical differences among groups (PC-1, CD-1, HD-1: 0.64 ± 0.50, 0.66 ± 0.54, 0.65 ± 0.61 pg/mL, respectively; all *P*>0.05). Postoperatively, significant increases in MTL concentrations were observed in all groups compared to baseline (PC-2: 0.72 ± 0.08 pg/mL; CD-2: 0.87 ± 0.08 pg/mL; HD-2: 0.90 ± 0.09 pg/mL; all *P* < 0.001). Comparisons among postoperative groups (ANCOVA-adjusted for baseline and time from surgery to blood draw) indicated significantly higher MTL levels in both CD-2 and HD-2 groups compared to PC-2 (*P* < 0.001 for both), but no significant difference between CD-2 and HD-2 groups (*P* = 0.951) (**Figure 2**). These findings suggest that *Bacillus coagulans* effectively enhances postoperative MTL secretion independently of dose escalation within the studied range. Across the cohort, the percentage rise in postoperative MTL was positively correlated with the magnitude of abdominal circumference reduction (*r* = 0.41, *P* < 0.01), indicating that greater motilin restoration aligned with greater relief of distension.

#### 3.2.2 Plasma 5-HT levels

Similar to MTL results, preoperative baseline levels did not differ significantly among groups (PC-1: 0.64 ± 0.69 ng/L; CD-1: 0.63 ± 0.10 ng/L; HD-1: 0.60 ± 0.99 ng/L; all *P*>0.05). Postoperatively, all groups exhibited significant increases compared to their respective baselines (PC-2: 0.74 ± 0.93 ng/L; CD-2: 0.87 ± 0.17 ng/L; HD-2: 0.86 ± 0.12 ng/L; all *P* < 0.001). Comparative analyses showed significantly higher postoperative 5-HT levels in CD-2 and HD-2 groups relative to PC-2 (*P* = 0.016 and *P* = 0.001, respectively), but again, no significant difference was detected between the two dosage groups (*P* = 1.000). Thus, *Bacillus coagulans* significantly promotes postoperative elevation of plasma 5-HT, potentially facilitating gastrointestinal motility recovery.

QC of ELISA assays indicated coefficients of variation of less than 5% across four plates, affirming assay precision and reliability for measuring plasma concentrations of both MTL and 5-HT. In summary, clinical findings robustly demonstrate that oral administration of *Bacillus coagulans* significantly shortens the recovery time of gastrointestinal function post gynecological laparoscopy. Furthermore, reductions in gastrointestinal adverse events and notable elevations of plasma MTL and 5-HT levels substantiate the efficacy and underlying biochemical mechanisms of *Bacillus coagulans* in promoting postoperative intestinal recovery.

### 3.3 Plasma metabolomic alterations associated with *Bacillus coagulans* administration

#### 3.3.1 Quality control and data reliability validation

**Figure 3:**
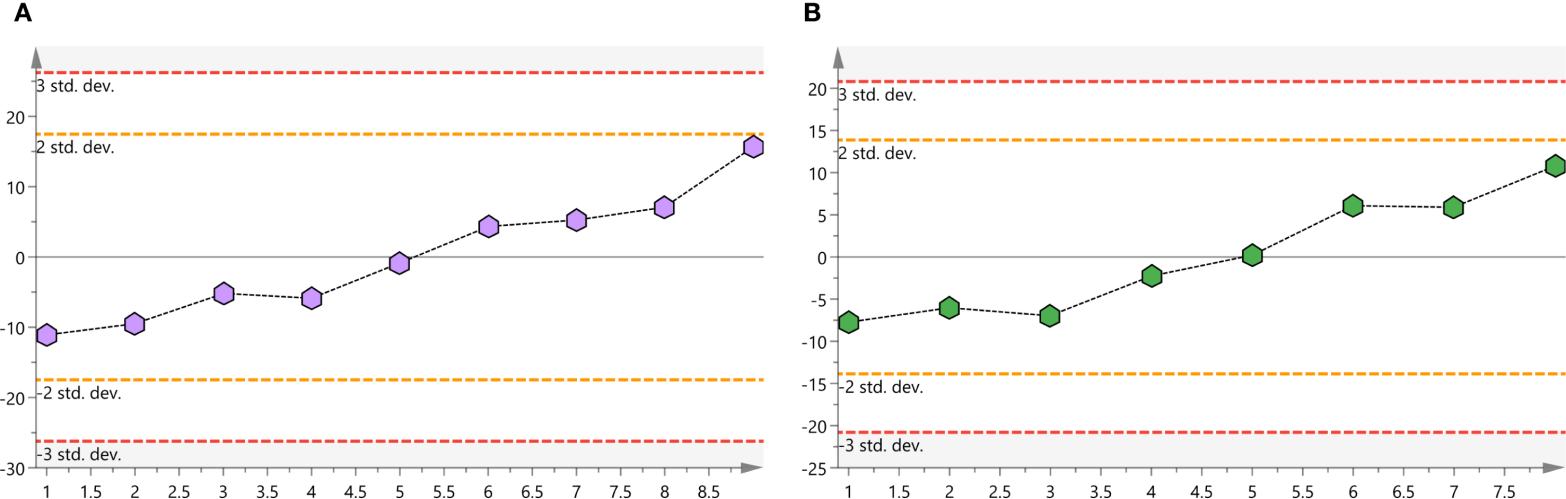
**(A)** Positive ion mode QC samples score plots. **(B)** Negative ion mode QC samples score plots. QC, quality-control.

Metabolomic profiling of patient plasma samples employed UHPLC-QTRAP™ mass spectrometry, with rigorous QC measures to validate analytical robustness. PCA analysis confirmed instrumental stability, as QC samples clustered tightly within the acceptable variation range (<2 standard deviations) and remained consistently within the 95% confidence interval (**Figures 3A, B**). Thus, analytical reproducibility and data integrity were ensured, allowing further in-depth metabolite comparison.

#### 3.3.2 Distinct metabolic signatures identified by multivariate analysis

**Figure 4:**
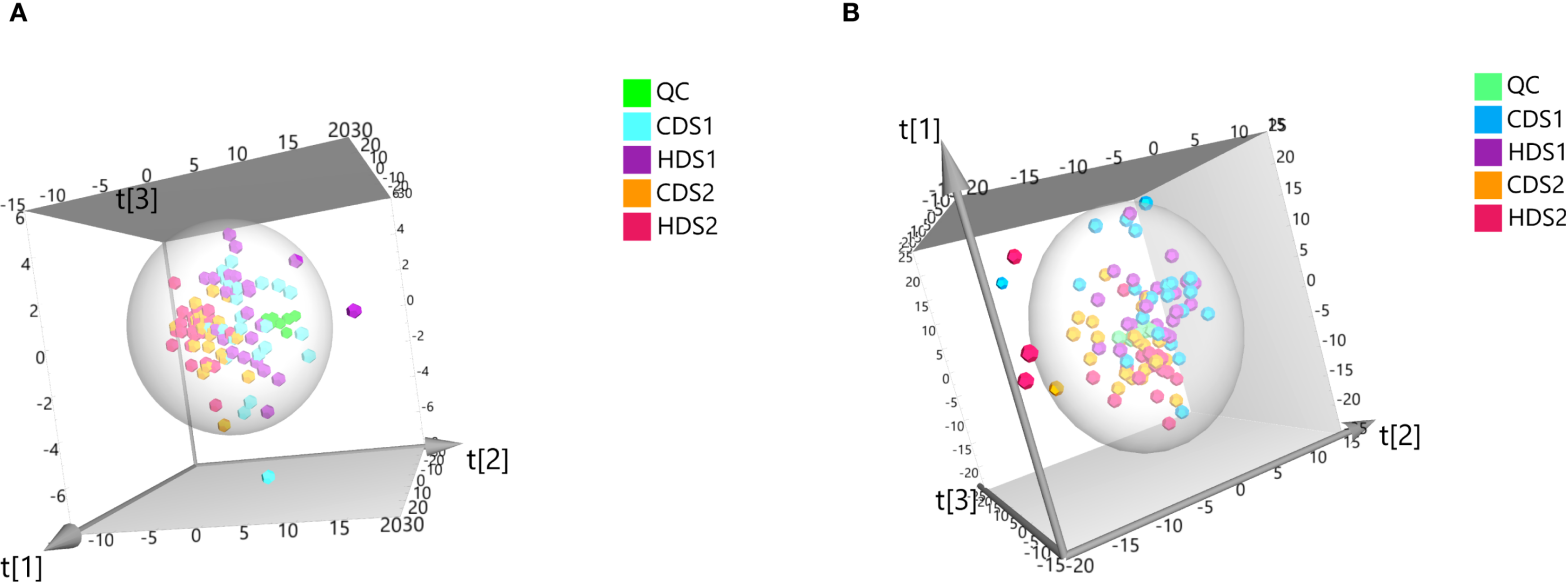
**(A)** Plot of PCA scores for QC, CDS1, HDS1, CDS2, and HDS2 in positive ion mode. **(B)** Plot of PCA scores for QC, CDS1, HDS1, CDS2, and HDS2 in negative ion mode. PCA, principal component analysis; QC, quality-control; CDS1, conventional; HDS1, high-dose; CDS2, conventional-dose; HDS2, high-dose.

**Figure 5:**
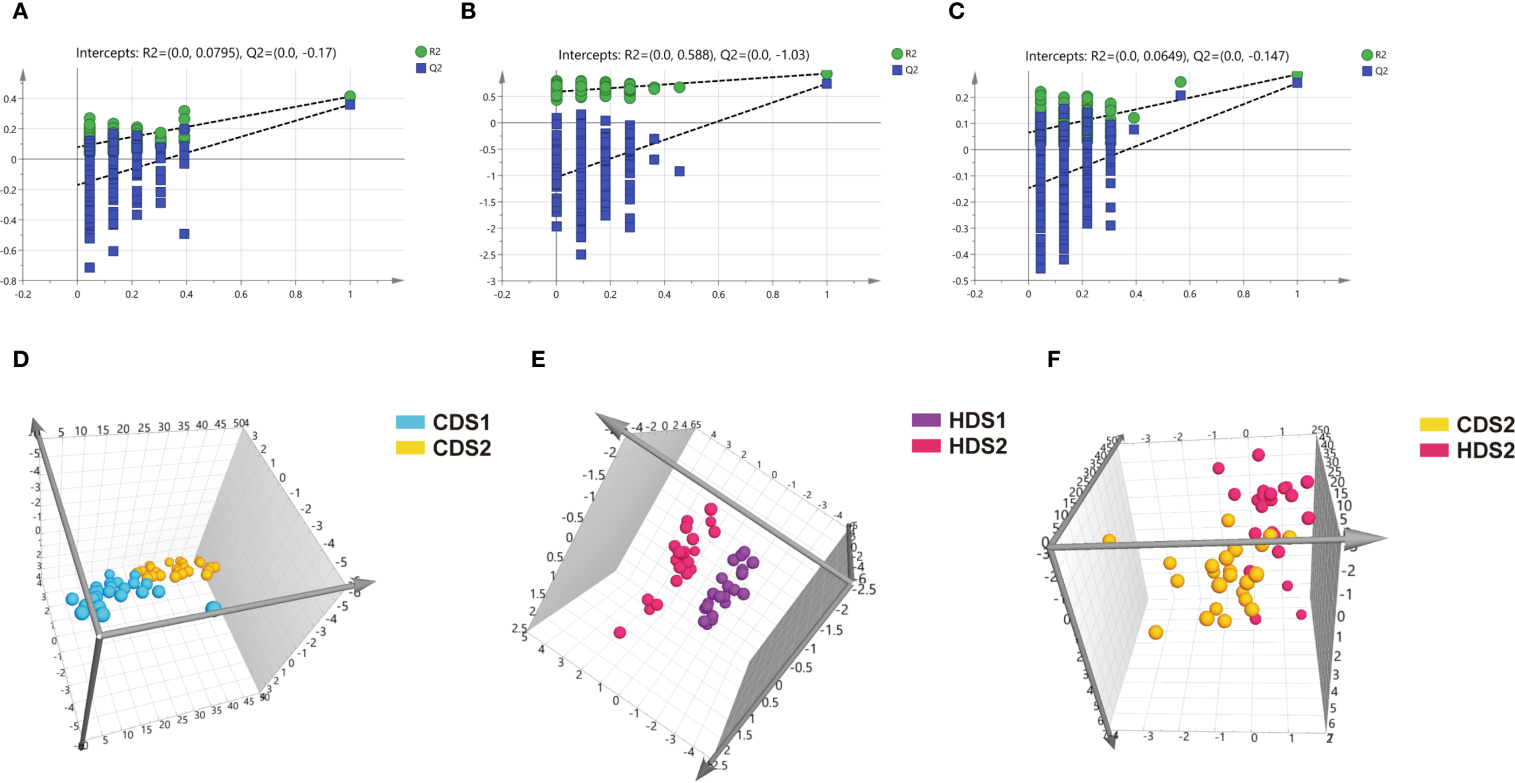
**(A–C)** Positive ion mode permutation test results for CDS1-CDS2 group, HDS1-HDS2 group, and CDS2-HDS2 group. **(D–F)** Positive ion mode OPLS-DA score plots for CDS1-CDS2 group, HDS1-HDS2 group, and CDS2-HDS2 group. CDS1, conventional dose—preoperative; CDS2, conventional dose—postoperative; HDS1, high dose—preoperative; HDS2, high dose—postoperative; OPLS-DA, orthogonal partial least squares discriminant analysis; *R²*, coefficient of determination; *Q²*, cross-validated predictive ability.

**Figure 6:**
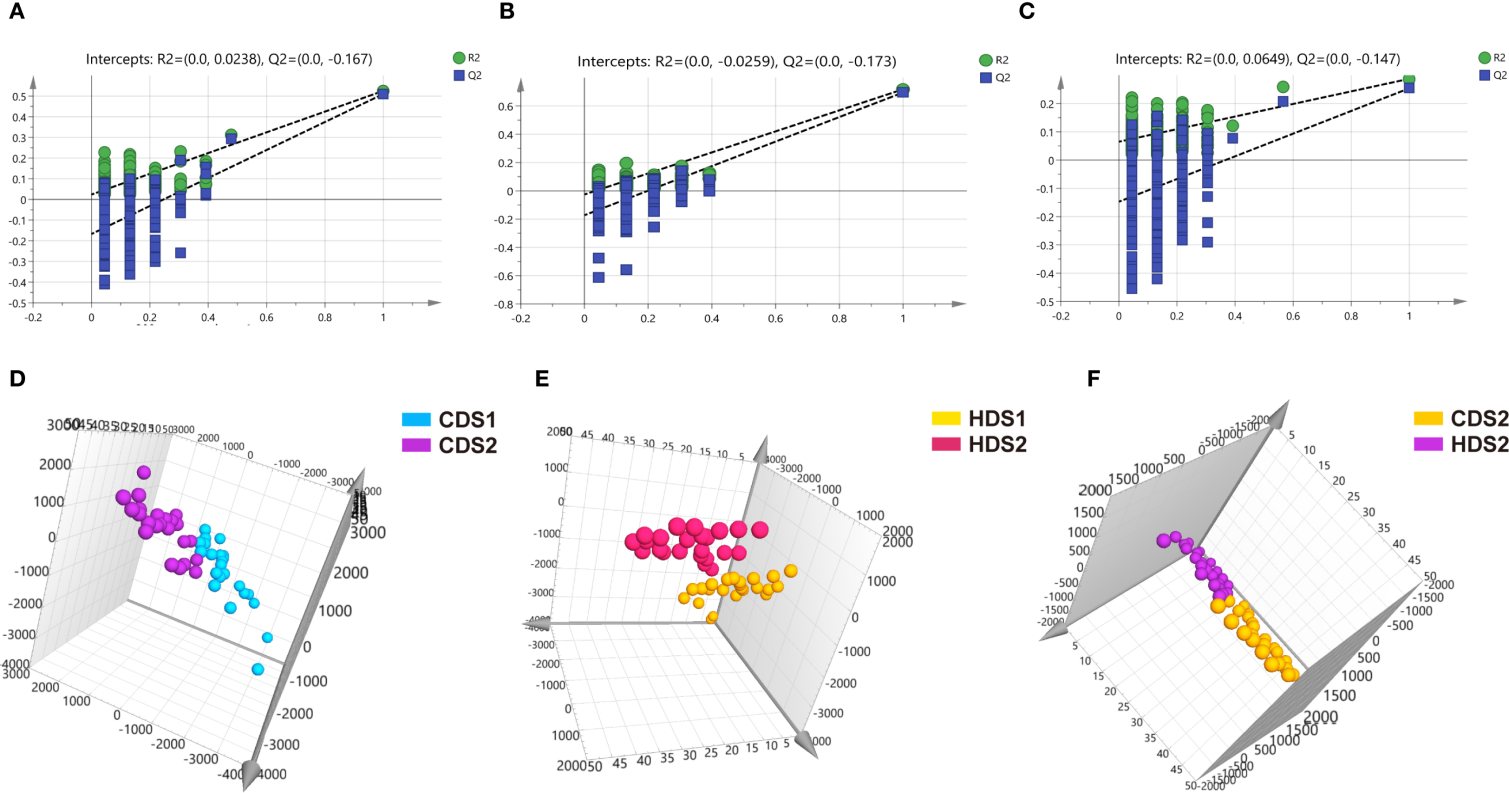
**(A–C)** Negative ion mode permutation test results for CDS1-CDS2 group, HDS1-HDS2 group, and CDS2-HDS2 group. **(D–F**) Negative ion mode OPLS-DA score plots for CDS1-CDS2 group, HDS1-HDS2 group, and CDS2-HDS2 group. CDS1, conventional dose—preoperative; CDS2, conventional dose—postoperative; HDS1, high dose—preoperative; HDS2, high dose—postoperative; OPLS-DA, orthogonal partial least squares discriminant analysis; *R²*, coefficient of determination (goodness of fit); *Q²*, cross-validated predictive ability.

PCA demonstrated clear clustering patterns indicating baseline similarity in metabolic profiles between the CD and HD groups before intervention (**Figures 4A, B**). Post-treatment, orthogonal partial least squares discriminant analysis (OPLS-DA) was utilized to characterize differences among groups effectively. OPLS-DA models demonstrated significant separations between pre- and post-treatment metabolic profiles within both CD and HD groups, indicating clear probiotic-induced metabolic alterations. Permutation tests further validated these models, excluding overfitting and confirming that differences were attributable specifically to *Bacillus coagulans* intervention rather than random variations (**Figures 5**, **6**). Additionally, OPLS-DA revealed subtle yet distinguishable metabolic differences between CD and HD groups post-treatment, indicating potential dose-related modulation effects. This analysis highlights that probiotic administration significantly reshapes patients’ plasma metabolic landscape in a dose-sensitive manner.

#### 3.3.3 Identification and characterization of differential metabolites

A total of 50 and 73 significantly altered metabolites were identified post-treatment within the CD and HD groups, respectively, compared with their pre-treatment levels (**Supplementary Table 1**). Comparative analysis between CD and HD post-treatment groups identified 18 significantly altered metabolites, suggesting dose-dependent metabolic shifts. Notably, 43 metabolites exhibited uniform change trends in both dosage groups: nine metabolites were consistently upregulated, and 34 metabolites consistently downregulated post-intervention. Among these, palmitoleic acid significantly increased in a dose-dependent manner, highlighting a potential key metabolite influenced by *Bacillus coagulans*. Conversely, metabolites such as Kyn and histidine consistently decreased, indicating probiotics’ possible anti-inflammatory and regulatory effects on amino acid metabolism. Kyn functions as an immunometabolite by engaging the aryl hydrocarbon receptor to modulate T-cell differentiation programs, while histidine serves as the precursor of microbially derived histamine that can amplify mucosal inflammatory responses (20). The alignment of these metabolic changes across dosage groups supports the validity of the observed trends, strengthening evidence that metabolic alterations are closely linked to the clinical efficacy of *Bacillus coagulans* rather than random occurrences or unrelated biological variability.

#### 3.3.4 Metabolic pathway enrichment analysis

**Figure 7:**
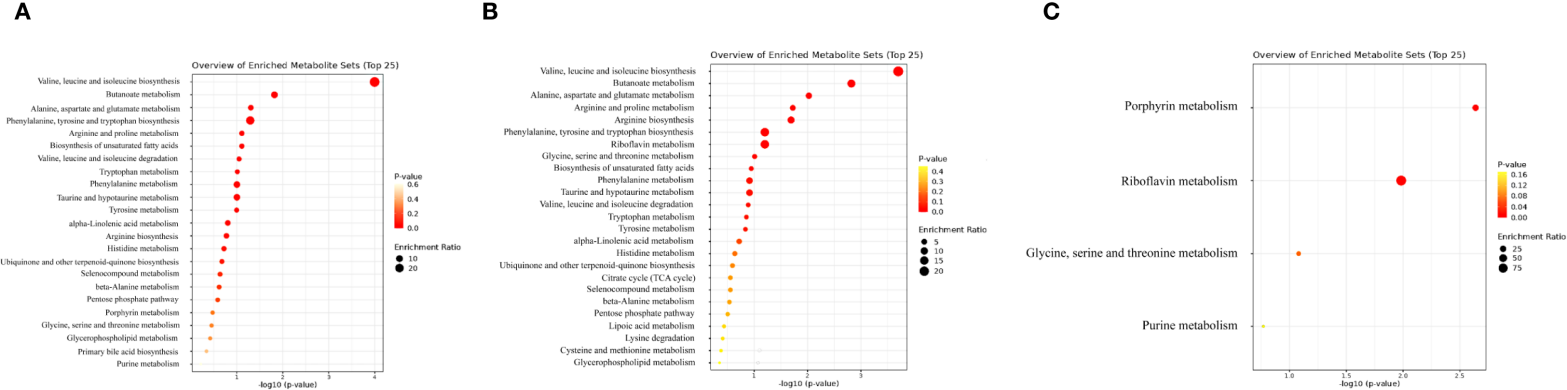
**(A)** KEGG metabolic pathway enrichment analysis of differential metabolites between CDS1 and CDS2. **(B)** KEGG metabolic pathway enrichment analysis of differential metabolites between HDS1 and HDS2. **(C)** KEGG metabolic pathway enrichment analysis of differential metabolites between CDS2 and HDS2. KEGG, Kyoto Encyclopedia of Genes and Genomes; CDS1, conventional dose—preoperative; CDS2, conventional dose—postoperative; HDS1, high dose—preoperative; HDS2, high dose—postoperative; TCA, tricarboxylic-acid (citrate) cycle.

**Figure 8:**
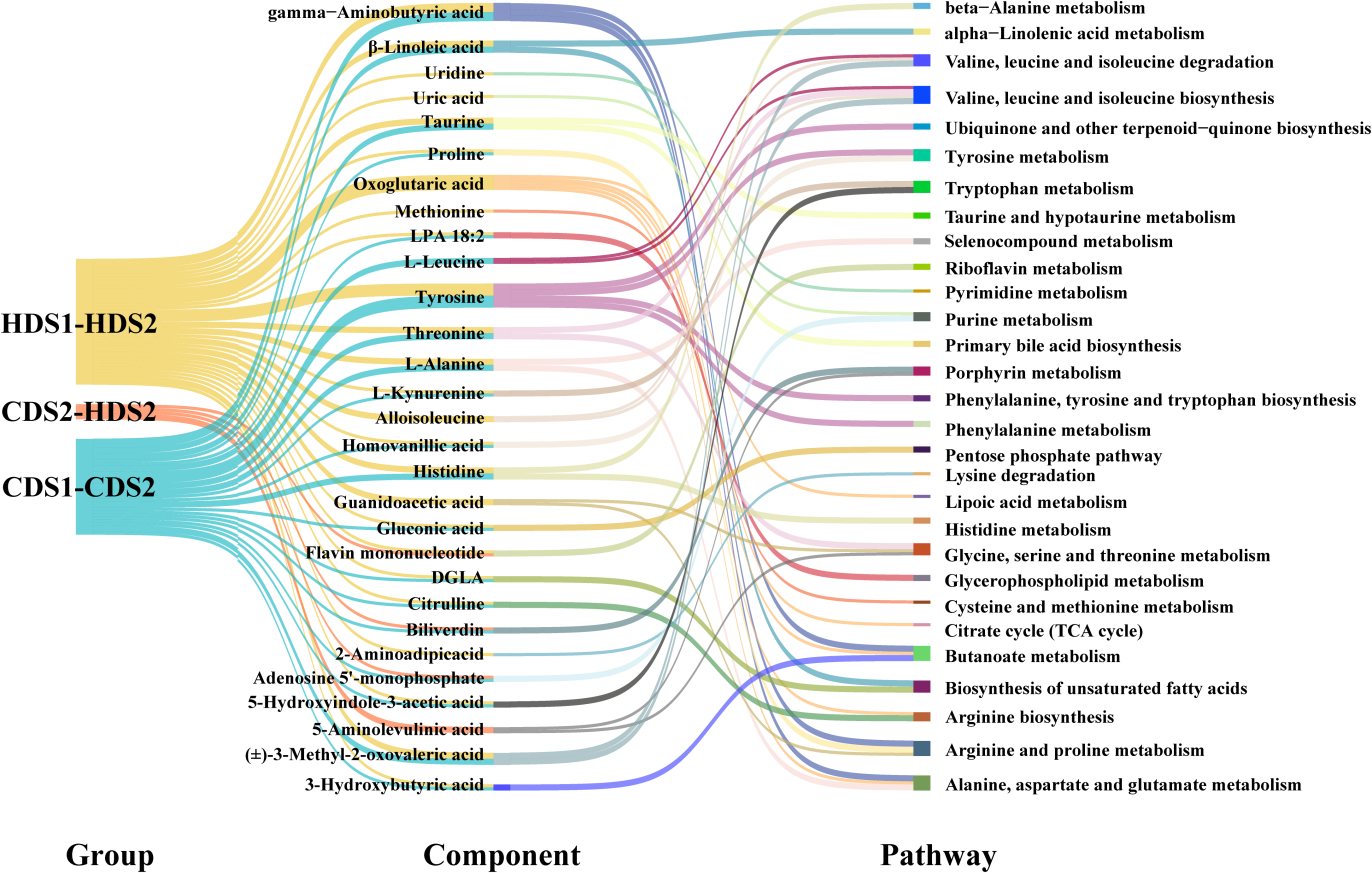
Differential metabolite component–metabolic pathway relationships between groups. LPA, lysophosphatidic acid; DGLA, dihomo-γ-linolenic acid; TCA cycle, tricarboxylic-acid cycle; CDS1/CDS2, conventional-dose pre/post; HDS1/HDS2, high-dose pre/post.

To understand the biological relevance of identified metabolites, KEGG enrichment analyses were performed using the MetaboAnalyst platform. Both CD and HD groups shared similar significantly enriched metabolic pathways post-treatment. The key pathways altered included tryptophan (Trp) metabolism, Kyn metabolism, tyrosine metabolism, histidine metabolism, phenylalanine metabolism, primary bile acid biosynthesis, purine metabolism, and short-chain fatty acid metabolism (**Figures 7A–C**, **8**). Among these pathways, Trp metabolism was notably modulated, which aligns closely with the observed increase in 5-HT plasma levels post-treatment. Enhanced 5-HT synthesis due to the shift from Kyn pathway metabolism towards the 5-HT pathway may contribute significantly to improved gastrointestinal motility and recovery observed clinically.

Tyrosine and histidine metabolism pathways, previously linked to inflammation and gut microbiome interactions, also showed marked changes post-intervention, suggesting a probiotic-mediated modulation of inflammatory responses and gut homeostasis. In addition, alterations in primary bile acid biosynthesis emphasize the probiotic’s role in lipid metabolism and gut microbiota regulation, potentially facilitating the re-establishment of intestinal barrier integrity postoperatively. Furthermore, short-chain fatty acid metabolism, particularly butyrate production, was positively regulated. Butyrate is known to strengthen intestinal barrier function, alleviate inflammation, and support gastrointestinal motility, all crucial aspects of postoperative recovery. Finally, subtle dose-related differences were observed in pathways related to glycine, serine, and threonine metabolism (GST), suggesting that higher probiotic dosage may confer additional metabolic benefits beyond baseline improvements seen at the CD. Such dose-dependent effects emphasize the importance of dosing strategies in probiotic interventions.

Collectively, these integrated metabolomic insights strongly indicate that *Bacillus coagulans* facilitates postoperative gastrointestinal recovery by comprehensively modulating key metabolic pathways. The consistency and reproducibility of these findings across both dose groups underscore the robust metabolic benefits imparted by *Bacillus coagulans*, providing a strong biochemical foundation for its clinical efficacy.

## 4 Discussion

Live *Bacillus coagulans* tablets deliver clear benefits. Spores survive gastric acid, reach the intestine, hydrolyze polysaccharides, and nourish resident *Bifidobacterium* spp. Friendly flora expand, bacteriocins restrain pathogens, microbial balance returns, toxin load falls, and postoperative ileus eases (21). Lactic acid and short−chain fatty acids acidify the lumen, activate colonic smooth muscle, accelerate peristalsis, shorten transit, and raise bowel sounds (22, 23). These observations collectively align with the postoperative improvements noted in our cohort. A schedule of six tablets the night before surgery and three every eight hours after anesthesia achieved full efficacy, standardized practice, cut waste, and reduced cost (18).

Motilin, a 22−residue peptide from duodenal and jejunal M cells, drives migrating motor complexes, speeds gastric emptying, and coordinates intestinal motility. Surgery and anesthetics suppress secretion (24); plasma concentration often drops within twenty−four hours after abdominal procedures, leading to distension and delayed postoperative bowel movement. Pre−operative levels in all groups were comparable. By postoperative day 1, motilin increased across all groups. Participants receiving *Bacillus coagulans* reported less bloating and achieved an earlier postoperative bowel movement. In the cohort, greater postoperative motilin restoration coincided with larger reductions in abdominal distension. Within the tested range, no significant between-dose difference was detected; accordingly, we interpret these findings mechanistically rather than quantitatively contrasting CD versus HD. This supports motilin restoration as one pathway by which *Bacillus coagulans* may facilitate recovery. Larger cohorts with longer follow-up could test whether sustained dosing maintains elevation and further shortens hospital stay.

Plasma 5−HT often rises slowly after gynecologic laparoscopy, even under balanced anesthesia (25). Fluctuating 5−HT and altered 5−HT turnover contribute to POGD (26). Preclinical *in vivo* studies demonstrate that 5-HT receptor–targeting agents shorten postoperative ileus and attenuate mucosal inflammation (27). The present trial confirmed a peri−operative shift in circulating 5−HT, mirroring earlier observations. SCFAs made by commensal bacteria up−regulate Trp hydroxylase, enhance 5−HT biosynthesis, and shield the epithelium in inflammatory bowel disease (28). More than ninety per cent of systemic 5−HT originates in enterochromaffin cells. Once released, it guides peristaltic reflexes, primes platelet aggregation, and modulates innate immunity. Microbial SCFAs trigger additional discharge from these cells (29, 30). Live *Bacillus coagulans* raises *Bifidobacterium* spp. and *Lactobacillus* spp. counts, organisms that stimulate enterochromaffin cells and stretch smooth muscle through SCFA production. The strain also fortifies tight junctions, helping normal 5−HT synthesis proceed. Every study arm displayed a postoperative upswing in plasma 5−HT. Mean elevation reached 13.51% in the PC, 27.59 % in the CD, and 30.23 % in the HD cohort. Tablets containing live *B. coagulans* delivered a steadier 5−HT profile and may have hastened functional recovery of the gut. Metabolomic shifts suggest engagement of AhR-linked Trp–Kyn signaling, FXR/TGR5 bile-acid pathways, and SCFA-driven Treg programs (31–34). Reduced histidine supports lower microbial histamine signaling. Gut-derived serotonin participates in EC-immune crosstalk, providing a plausible link between motility and mucosal immunity (35).

Untargeted metabolomics identified 50 differential metabolites when pre− and post−operative samples from the CD group were compared, 73 in the corresponding HD comparison, and 18 between the two post−operative dose groups. Among the first two comparisons, 43 compounds shifted in the same direction, nine rising and 34 falling. Parallel movement across separate doses suggests a drug−related effect rather than random fluctuation. Dose escalation produced coherent changes in three metabolites that also reached statistical significance. Palmitoleic acid, a monounsaturated lipokine tied to epithelial repair, increased in both dose groups and rose further in HD than in CD. Two lysophospholipids, [2−(1,2−dihydroxyethoxy)−3−[2−(dimethylamino)ethoxy−hydroxyphosphoryl]oxypropyl] hexadecanoate and LPE 20:2, declined after treatment, with a deeper drop in HD. The higher dose therefore amplified the primary pharmacodynamic signal. When multiple metabolites track dose in a unified manner, a mechanistic relationship is likely; this gradient helps locate an optimal therapeutic window and supplies biochemical targets for future monitoring.

Trp feeds two enzymatic routes. One yields 5−HT through tryptophan hydroxylase and ultimately melatonin. The other, catalyzed by indoleamine 2,3−dioxygenase 1, funnels Trp into Kyn and related products (36, 37). Under baseline conditions, less than one per cent of Trp enters the 5−HT pathway. Kyn levels vary with hormonal status (38); this confounder was absent because none of the participants were menstruating. Patients with irritable bowel syndrome exhibit higher free Trp, lower serum Kyn, and greater microbial degradation along the Kyn branch (29). Released 5−HT binds local neuronal receptors, activates intrinsic afferents, or enters the portal flow where platelets absorb it. Re−release into the lumen targets mucosal 5−HT_3_, 5−HT_1_P, or 5−HT₇ receptors, triggers migrating motor complexes, and further shapes Trp metabolism (39). Exogenous Trp eases DSS colitis by adjusting HTR receptor gene expression (40). The immune circuit that links these changes to dysbiosis continues to unfold. Post−operative meals followed a uniform non−dairy light protocol (17), limiting dietary impact on Trp and 5−HT flux. Tyrosine interacts with the microbiome as well. Supplementation in a graft−versus−host model boosted beneficial genera and reduced diarrhea (41). Tyrosine concentrations fall during intestinal inflammation, a trend echoed by our metabolic screen.

Anesthesia, opiate analgesia, and tissue handling provoke adrenergic bursts and cytokine release that disrupt enteric neuron firing. Perfusion shifts lower epithelial oxygen, weaken strict anaerobes, and divert fermentation away from butyrate, a driver of motility and barrier repair. Early microbial support with *B. coagulans* restores SCFA ratios and sustains butyrate−producing taxa. Metabolites detected by high−resolution mass spectrometry clustered into lipid mediators, amino−acid derivatives, and energy intermediates. Triacylglycerol−50:2 fell after both doses, compatible with faster propulsion because prolonged stasis allows lipolysis and reabsorption. Ornithine accumulated, reflecting polyamine synthesis needed for epithelial restitution. Betaine declined, implying use as a methyl donor in membrane repair. Dose stratification carried clinical value. The high dose required one extra administration yet produced stronger metabolic adjustment without adverse effects; adherence topped 95%. Palmitoleic acid elevation hints at recovery of epithelial lipid synthesis capacity. Concurrent reduction in injury−linked lysophospholipids signals less membrane stress. Certain Kyn derivatives activate the aryl hydrocarbon receptor, steering T−cell fate. Tracking this branch may reveal an immunologic fingerprint of effective probiotic therapy. Untargeted spectra limit quantification. Future work should add isotope dilution, link chemical shifts to scintigraphic transit, and sample mucosal cytokines. Even with these gaps, the trajectory is clear: early microbial support rebuilds a biochemical environment that favors swift recovery of coordinated motility and barrier integrity.

Subgroup analyses consistently implicated the GST metabolism axis, implying a dose-responsive pharmacodynamic focus of live *Bacillus coagulans* on GST turnover. Roughly 71% of dietary threonine fuels intestinal mucin synthesis; scarcity impairs barrier integrity and slows digestion (42). In dextran−sulfate−sodium colitis, GST disruption alters acetyl−CoA supply to the tricarboxylic−acid cycle, weakening cellular energy. A *Coix lacryma–Epiphyllum* compound restored dozens of metabolites by normalizing GST flux and eased inflammation in that model (43). The parallel observed here suggests a shared metabolic checkpoint between herbal and probiotic interventions.

Administration of live spores shifted tryptophan derivatives toward baseline and modulated phenylalanine plus GST pathways. Such coordination matches earlier work linking probiotic therapy to concurrent control of Kyn, indole, and GST networks. The response included a decline in circulating histidine, a signal that histidine decarboxylation within the lumen, a source of pro−inflammatory histamine, had slowed. Combined data point to a multi−node mechanism: live *B. coagulans* expands beneficial genera, suppresses histamine−forming taxa, and harmonizes amino−acid metabolism, thereby calming mucosal immunity and accelerating motility.

Bile acids operate as chemical go−betweens for host and microbiota through Farnesoid X and TGR5 receptors (44). Their enterohepatic circulation governs lipid absorption and limits pathogen growth (45). Present profiles reveal a comparable adjustment: primary bile−acid output fell after probiotic ingestion, hinting that toxin−rich secondary species dropped as substrates dwindled. The shift may lessen epithelial stress and favor quick resolution of postoperative dysmotility.

POGD often follows an upswing of facultative pathogens that bloom once oxygen tension changes during surgery (46). Depressed nucleotide metabolism in this setting alters purine flux within the microbial community (47). Restricting purines hampers replication, translation, and chaperone folding in harmful strains, nudging the ecosystem back toward balance. Our data reproduced this trajectory: nucleotide precursors fell, suggesting that *B. coagulans* tilted resource competition away from opportunists, limited pathogen survival, and indirectly restored coordinated propulsion of the bowel.

Fermentation of dietary polysaccharides yields butyrate, a short−chain fatty acid that energizes colonocytes, tightens junctions, and up−regulates tryptophan hydroxylase 1, boosting peripheral 5−HT and peristalsis (48). Butyrate also mitigates oxidative stress and repairs barrier lesions through AMPK−driven mitophagy (49). Metabolomic patterns in this trial showed enhanced butyrate generation after probiotic dosing, consistent with recovery of key anaerobes. Elevated butyrate aligns with faster return of bowel sounds and earlier postoperative bowel movement noted clinically, reinforcing the view that metabolite−guided support of the epithelium underpins the therapeutic effect.

Observed biochemical and symptomatic gains outline a credible option for preventing or treating POGD in gynecologic laparoscopy. Future work should enlarge the cohort, include multiple centers, and stratify by surgical duration, antibiotic exposure, and baseline microbiota. A randomized design with isotope−labelled tracers could map real−time flux through GST and bile−acid circuits, verifying causality. Safety signals remained benign in this pilot, yet extended surveillance will confirm tolerability at higher doses or longer courses. Such refinement may position live *B. coagulans* tablets as a cost−effective adjunct that secures quicker gastrointestinal recovery and shorter hospital stays.

## 5 Conclusion

The trial was a randomized controlled double-blind clinical trial that recruited gynecological laparoscopic surgery patients, gave PC to the placebo group, and gave the drug intervention group to take live *Bacillus coagulans* tablets during the period from preoperative to the first postoperative anal defecation. The results showed that the drug could safely and effectively shorten the recovery time of postoperative intestinal function, and it had a promoting and regulating effect on the secretion of MTL and 5-HT in plasma, so that they could return to normal as soon as possible, and play a physiological role in promoting gastric emptying and intestinal peristalsis. Metabolomics analysis showed that the drug can effectively maintain intestinal homeostasis, and its mechanism may be related to the regulation of amino acid metabolism, Kyn metabolism, tyrosine metabolism, histidine metabolism, phenylalanine metabolism, arginine metabolism, primary bile acid biosynthesis, and the biosynthesis of tyrosine and tryptophan, and at the same time, dose changes will cause the metabolism of GST, riboflavin metabolism, porphyrin metabolism and purine metabolism. Pathway function changes. In conclusion, the indicated dose of *Bacillus coagulans* tablets can safely and effectively improve postoperative bowel function in gynecologic laparoscopic patients.

## Data Availability

The original contributions presented in the study are included in the article/**Supplementary Material**. Further inquiries can be directed to the corresponding authors.
