# Supplementary material for: Integrated metabolomic insights into the mechanism of Bacillus coagulans in enhancing intestinal recovery following gynecological laparoscopic surgery: a randomized clinical trial
Source: Front Immunol. 2025 Sep 26;16:1630447. doi: 10.3389/fimmu.2025.1630447 (PMC12511109; doi:10.3389/fimmu.2025.1630447)
Supplement: Supplementary file 1 [file Table1.docx]

Supplementary Table 1. Differential metabolite information and trends between groups (where ↑ represents up-regulation of differential metabolite abundance and ↓ represents down-regulation of differential metabolite abundance; *, ** represent statistical differences of *P* < 0.05 and *P* < 0.01 between CDS1-CDS2 groups, respectively.; #, ## represent statistical differences of *P* < 0.05 and *P* < 0.01 between HDS1-HDS2 groups, respectively.; ▲, ▲▲represent statistical differences of *P* < 0.05 and *P* < 0.01 between CDS2-HDS2 groups, respectively. \ represents no statistical difference in a subgroup comparison for this compound).

| Number | Compound name | Molecular formula of the compound | High-resolution data | CDS2/CDS1 Group trends | | HDS2/HDS1 Group trends | | HDS2/CDS2 Group trends | |
| --- | --- | --- | --- | --- | --- | --- | --- | --- | --- |
| 1 | Acar 10:1 | C_17_H_32_NO_4_ | 314.23242,85.0311 | ↑ | \ | ↑ | # | ↓ | ▲▲ |
| 2 | Pro-Leu | C_11_H_20_N_2_O_3_ | 229.1529,132.1019,89.0969,70.0672 | ↑ | ** | ↑ | ## | ↓ | \ |
| 3 | Tyrosine | C_9_H_11_NO_3_ | 182.0835,136.0757,123.0443,119.0497,95.0498,91.0557 | ↑ | \ | ↓ | # | ↓ | \ |
| 4 | Citrulline | C_6_H_13_N_3_O_3_ | 176.1032,159.0756,141.0655,133.0968,115.0865,113.0710 | ↓ | * | ↓ | ## | ↑ | \ |
| 5 | DL-proline | C_5_H_9_NO_2_ | 116.0708,70.0657,72.0813,68.0501 | ↓ | ** | ↓ | ## | ↓ | \ |
| 6 | 2-(5,8-Tetradecadienyl)-cyclobutanone | C_18_H_30_O | 263.2361,245.2256,219.2107,189.1635,179.1787,177.1653,165.1632,163.1482,151.1482,149.1320,137.1327,135.1178,123.1163,109.1020,97.1016,69.0701 | ↑ | * | ↑ | # | ↑ | \ |
| 7 | (5Z,8Z,11Z,14Z)-16-hydroxyicosa-5,8,11,14-tetraenoic acid(16-HETE) | C_20_H_32_O_3_ | 321.23,303.13 | ↑ | \ | ↑ | ## | ↑ | ▲ |
| 8 | Acar 5:0 | C_12_H_23_NO_4_ | 246.1698,85.0289 | ↓ | ** | ↓ | ## | ↓ | \ |
| 9 | Vaccenic acid | C_18_H_34_O_2_ | 283.2635,265.2491,247.2382,227.2004,163.1459,149.1321 | ↑ | ** | ↑ | ## | ↑ | \ |
| 10 | Palmitoleic acid | C_16_H_30_O_2_ | 255.2317,237.2216,219.2113,109.1015,97.1013,57.0730 | ↑ | ** | ↑ | ## | ↑ | ▲ |
| 11 | Erucamide | C_22_H_43_NO | 338.3408,321.3186,303.3012,163.1481,149.1324,83.0855 | ↑ | \ | ↑ | # | ↑ | \ |
| 12 | Oleamide | C_18_H_35_NO | 282.2791,265.2517,247.2407 | ↑ | \ | ↑ | # | ↑ | ▲ |
| 13 | Palmitic amide | C_16_H_33_NO | 256.2647,88.0780,95.0881,57.0741,55.0586 | ↓ | \ | ↑ | # | ↑ | ▲ |
| 14 | LPC(16:0) | C_24_H_50_NO_7_P | 496.3364,478.3277,184.0729,104.1078 | ↓ | ** | ↓ | ## | ↑ | \ |
| 15 | 5-Hydroxyindole-3-acetic acid | C_10_H_9_NO_3_ | 192.0653,174.0547,146.0595,128.0491,118.0860 | ↓ | ** | ↓ | ## | ↑ | \ |
| 16 | 3-Indolepropionic acid | C_11_H_11_NO_2_ | 190.08,130.06 | ↓ | \ | ↓ | ## | ↓ | ▲ |
| 17 | β-Linoleic acid | C_18_H_32_O_2_ | 281.2486,263.2370,245.2263,235.2478,221.2254,207.2159,97.1021 | ↑ | ** | ↑ | ## | ↑ | \ |
| 18 | Adenosine 5'-monophosphate | C_10_H_14_N_5_O_7_P | 348.07,136.06 | ↑ | ** | ↓ | \ | ↓ | ▲ |
| 19 | 4-Hydroxyquinoline | C_9_H_7_NO | 146.0596,128.0494,101.0396,77.0387 | ↓ | \ | ↓ | # | ↓ | \ |
| 20 | Abscisic acid | C_15_H_20_O_4_ | 265.1433,247.1334,229.1222,203.1061,201.1272,187.1114,135.0813,123.0809 | ↑ | ** | ↑ | ## | ↑ | \ |
| 21 | Piperidine | C_5_H_11_N | 86.09,69.07 | ↑ | \ | ↓ | # | ↓ | ▲▲ |
| 22 | Biliverdin | C_33_H_34_N_4_O_6_ | 583.25493,297.1248 | ↑ | ** | ↑ | \ | ↓ | ▲▲ |
| 23 | DGLA | C_20_H_34_O_2_ | 307.26338,67.058 | ↑ | * | ↑ | ## | ↑ | \ |
| 24 | 3,4-Epoxy-6,9-octadecadiene | C_18_H_32_O | 265.2535,247.2429,163.1472 | ↑ | ** | ↑ | ## | ↑ | \ |
| 25 | Farnesylacetone | C_18_H_30_O | 263.23669,245.2275 | ↑ | ** | ↑ | ## | ↑ | \ |
| 26 | Phe-Pro | C_14_H_18_N_2_O_3_ | 263.13887,116.0706,70.0673 | ↓ | \ | ↑ | \ | ↑ | ▲ |
| 27 | Nicotyrine | C_10_H_10_N_2_ | 159.09138,132.0815 | ↓ | \ | ¯ | # | ¯ | \ |
| 28 | Homocycloleucine | C_7_H_13_NO_2_ | 144.1018,58.0691 | ↓ | \ | ↑ | ## | ↑ | \ |
| 29 | D-Pipecolinic acid | C_6_H_11_NO_2_ | 57.0699,84.0808,86.0964,94.0651,112.0757,130.0863 | ↓ | * | ↓ | ## | ↑ | \ |
| 30 | Uridine | C_9_H_12_N_2_O_6_ | 245.18554,69.07071 | ↑ | \ | ↑ | # | ↑ | \ |
| 31 | Flavin mononucleotide | C_17_H_21_N_4_O_9_P | 457.34888,439.1154,243.0883,91.0548,77.0381 | ↓ | \ | ↑ | ## | ↑ | ▲▲ |
| 32 | gamma-Aminobutyric acid | C_4_H_9_NO_2_ | 104.107,58.066 | ↓ | ** | ↓ | # | ↓ | \ |
| 33 | Guanidoacetic acid | C_3_H_7_N_3_O_2_ | 118.0617,101.0359,76.0403,72.0569 | ↓ | \ | ↓ | # | ↓ | \ |
| 34 | 5-Aminolevulinic acid | C_5_H_9_NO_3_ | 132.10174,69.07036 | ↑ | \ | ↓ | \ | ↓ | ▲▲ |
| 35 | LPC 20:1 | C_28_H_56_NO_7_P | 550.38,184.07 | ↓ | ** | ↓ | ## | ↑ | \ |
| 36 | PC(18:0/0:0) | C_26_H_54_NO_7_P | 524.3698,506.3601,184.0731,104.1073 | ↓ | * | ↓ | ## | ↑ | \ |
| 37 | L-Alanine | C_3_H_7_NO_2_ | 90.0545,55.0174,44.0495 | ↓ | * | ↓ | # | ↑ | \ |
| 38 | Methionine | C_10_H_20_N_2_O_3_S_2_ | 150.0583,104.0508,87.0257,84.0443,61.0104,56.0492 | ↓ | \ | ↓ | # | ↓ | \ |
| 39 | Tyrosine | C_9_H_11_NO_3_ | 182.0812,136.0764,123.0436,119.0497,91.05699,95.0501 | ↓ | ** | ↓ | ## | ↓ | \ |
| 40 | Proline | C_6_H_6_O_3_ | 116.0706,70.0649,43.0553 | ↓ | ** | ↓ | ## | ↓ | \ |
| 41 | Threonine | C_4_H_9_NO_3_ | 120.0655,103.0390,102.0541,84.0443,76.0390,75.0446,74.0595 | ↓ | * | ↓ | ## | ↓ | \ |
| 42 | L-Citrulline | C_6_H_13_N_3_O_3_ | 176.1032,159.0756,141.0655,133.0968,115.0865,113.0710 | ↓ | ** | ↓ | # | ↓ | \ |
| 43 | L-Kynurenine | C_10_H_12_N_2_O_3_ | 209.0921,192.0661,120.0465,118.0669 | ↓ | ** | ↓ | # | ↓ | \ |
| 44 | Uric acid | C_5_H_4_N_4_O_3_ | 167.0219,124.0154,96.0215 | ↓ | \ | ↓ | # | ↓ | \ |
| 45 | P-cresol sulfate | C_7_H_8_O_4_S | 187.008,107.05 | ↓ | \ | ↓ | ## | ↓ | \ |
| 46 | 4-Hydroxybenzenesulfonic acid | C_6_H_6_O_4_S | 172.992,93.03 | ↓ | ** | ↓ | \ | ↓ | \ |
| 47 | Taurine | C_2_H_7_NO_3_S | 124.0070,79.9584 | ↓ | ** | ↓ | ## | ↓ | \ |
| 48 | LPE 20:2 | C_25_H_48_NO_7_P | 504.31,279.23 | ↓ | ** | ↓ | ## | ↓ | ▲▲ |
| 49 | [2-(1,2-Dihydroxyethoxy)-3-[2-(dimethylamino)ethoxy-hydroxyphosphoryl]oxypropyl] hexadecanoate | C_25_H_52_NO_9_P | 540.3372,480.3146,255.2353,152.9919 | ↓ | ** | ↓ | ## | ↓ | ▲▲ |
| 50 | Phosphoric acid | H_3_O_4_P | 96.96,79.96 | ↑ | \ | ↓ | ## | ↓ | \ |
| 51 | Suberic acid | C_8_H_14_O_4_ | 201.1144,183.1049,157.1242,139.1136 | ↓ | * | ↓ | ## | ↓ | \ |
| 52 | alpha-Ketoglutarate | C_5_H_6_O_5_ | 145.0137,101.0262 | ↓ | \ | ↓ | ## | ↓ | \ |
| 53 | (±)-3-Methyl-2-oxovaleric acid | C_6_H_10_O_3_ | 129.0568,101.0614,85.0713 | ↓ | \ | ↓ | ## | ↓ | \ |
| 54 | Citraconic acid | C_5_H_6_O_4_ | 129.0188,85.0308 | ↓ | ** | ↓ | ## | ↓ | \ |
| 55 | 2-Aminoadipicacid | C_6_H_11_NO_4_ | 160.0610,116.0322,98.0242 | ↓ | \ | ↓ | ## | ↓ | \ |
| 56 |  |  |  |  |  |  |  |  |  |
| 57 | Indoxyl sulfate | C_8_H_7_NO_4_S | 212.0024,132.0449,80.9651,77.0400 | ↓ | ** | ↓ | ## | ↓ | \ |
| 58 | 2-Hydroxy-3-methylbutyric acid | C_5_H_10_O_3_ | 117.0503,99.0404,75.0101,71.0498 | ↓ | * | ↓ | ## | ↓ | \ |
| 59 | Histidine | C_6_H_9_N_3_O_2_ | 154.0636,137.0364,110.0740,93.0464,81.0478,72.0113 | ↓ | ** | ↓ | ## | ↓ | \ |
| 60 | LPA 18:2 | C_21_H_39_O_7_P | 433.24,152.99 | ↓ | ** | ↓ | ## | ↓ | \ |
| 61 | LPI 20:4 | C_29_H_49_O_12_P | 619.289,303.233 | ↑ | \ | ↓ | ## | ↓ | ▲▲ |
| 62 | Creatinine | C_4_H_7_N_3_O | 112.2,78.8 | ↓ | \ | ↓ | ## | ↓ | \ |
| 63 | 2-Hydroxyisocaproic Acid | C_6_H_12_O_3_ | 131.0709,85.0650,69.0338 | ↓ | \ | ↓ | # | ↓ | \ |
| 64 | CMPF | C_12_H_16_O_5_ | 239.09,195.1 | ↑ | ** | ↓ | \ | ↓ | \ |
| 65 | FAHFA 36:1 | C_36_H_68_O_4_ | 563.50181,281.2479 | ↓ | ** | ↓ | # | ↓ | \ |
| 66 | FA 20:0 | C_20_H_40_O_2_ | 311.29644,183.0112 | ↓ | \ | ↓ | \ | ↑ | ▲▲ |
| 67 | Hippuric acid | C_9_H_9_NO_3_ | 178.05232,134.0606 | ↓ | ** | ↓ | ## | ↓ | \ |
| 68 | N-Acetyl-serine | C_5_H_9_NO_4_ | 146.0478,116.0370,74.0263 | ↓ | \ | ↓ | ## | ↓ | \ |
| 69 | Homovanillic acid | C_9_H_10_O_4_ | 181.0518,163.0397,137.0616,122.0369 | ↓ | * | ↓ | ## | ↓ | \ |
| 70 | Gluconic acid | C_6_H_12_O_7_ | 195.0521,129.0200,75.0106 | ↓ | ** | ↓ | ## | ↓ | \ |
| 71 | Indolelactic acid | C_11_H_11_NO_3_ | 204.0686,186.0583,158.0619,142.0675,128.0514,116.0521 | ↓ | ** | ↓ | ## | ↓ | \ |
| 72 | 3,4-Dihydroxyhydrocinnamic acid | C_9_H_10_O_4_ | 181.0517,137.0610 | ↓ | \ | ↓ | # | ↓ | \ |
| 73 | Salicylic acid | C_7_H_6_O_3_ | 137.02577,93.0357 | ↓ | ** | ↓ | ## | ↓ | \ |
| 74 | 2-Hydroxyhexadecanoic acid | C_16_H_32_O_3_ | 271.2298,253.2190,235.2202 | ↓ | ** | ↓ | # | ↓ | \ |
| 75 | PE(17:0/0:0) | C_22_H_46_NO_7_P | 466.3,296.25 | ↓ | ** | ↓ | ## | ↓ | \ |
| 76 | Oleoyl LPA | C_21_H_41_O_7_P | 435.25039,255.2319 | ↓ | \ | ↓ | ## | ↓ | \ |
| 77 | Alloisoleucine | C_6_H_13_NO_2_ | 44.3500,99.8978 | ↓ | \ | ↓ | # | ↓ | \ |
| 78 | L-Leucine | C_6_H_13_NO_2_ | 44.9000,66.1000,71.9000,78.7000,81.9000,84.2000,130.0000 | ↓ | * | ↓ | \ | ↑ | \ |
| 79 | FA 21:0 | C_21_H_42_O_2_ | 307.0000,324.0000,325.0000,326.0000 | ↑ | * | ↑ | \ | ↓ | \ |
| 80 | Dodecylbenzenesulfonic acid | C_18_H_30_O_3_S | 325.18,183.01 | ↓ | \ | ↑ | \ | ↑ | ▲ |
| 81 | 3-Hydroxybutyric acid | C_4_H_8_O_3_ | 103.0395999,59.0141519 | ↑ | \ | ↑ | ## | ↓ | \ |
| 82 | 9,12,13-TriHOME | C_18_H_34_O_5_ | 59.0139,71.0866,83.0866,99.0815,101.0972,129.0219,131.1087,141.0921,143.1087,145.1234,157.1234,169.1234,181.1234,183.1391,185.1183,187.1340,199.1340,209.1183,211.1340,213.1496,227.1289,229.1445,241.1445,257.1394,267.1330,285.2345,293.2122,311.2228,329.2333 | ↓ | * | ↓ | ## | ↑ | \ |
| 83 | LPG 18:1 | C_24_H_47_O_9_P | 509.2885 | ↓ | ** | ↓ | ## | ↓ | \ |
| 84 | 2-deoxy-d-glucose | C_6_H_12_O_5_ | 163.06319,101.0252 | ↓ | \ | ↓ | # | ↑ | \ |
| 85 | Phenylacetylglycine | C_10_H_11_NO_3_ | 192.0682,174.0538,148.0776,91.0548,72.0249,56.0156 | ↑ | * | ↓ | \ | ↓ | ▲▲ |
